# Small-angle X-ray scattering intensity of multiscale models of spheroids

**DOI:** 10.1107/S1600576722011359

**Published:** 2023-02-01

**Authors:** Pascal Duchêne, Séverine Humbert, Loïc Sorbier, Maxime Moreaud

**Affiliations:** a IFP Energies nouvelles, Rond-point de l’échangeur de Solaize, BP 3, 69360 Solaize, France; b MINES ParisTech, PSL-Research University, CMM, 35 rue Saint Honoré, 77305 Fontainebleau, France; Argonne National Laboratory, USA

**Keywords:** small-angle X-ray scattering, Boolean models, multiscale aggregates, catalysts, supports, spheroids

## Abstract

The microstructure of heterogeneous catalysts often consists of multiscale aggregates of nanoparticles, some of which are highly anisotropic. Therefore, small-angle X-ray scattering, in classical or anomalous mode, is a valuable tool to characterize this kind of material. Yet, the classical exploitation of the scattered intensities through form and structure factors or by means of Boolean models of spheres is questionable. Here, it is proposed to interpret the scattered intensities through the use of multiscale Boolean models of spheroids. The numerical procedure to compute scattered intensities of such models is given and then validated on asymptotic diluted Boolean models, and its applicability is demonstrated for the characterization of alumina catalyst supports.

## Abstract 2

Small-angle X-ray scattering intensity is simulated for multiscale Boolean models of spheroids.

## 1. Introduction

Heterogeneous catalysts are of primary importance in the production of chemicals (Dingerdissen *et al.*, 2008 ▸). These catalysts often consist of nanoparticles (the active phase) deposited on a nanostructured support with scales ranging from nanometres to micrometres (Weckhuysen, 2009 ▸). The support or the active phase may be viewed as a collection of randomly stacked grains. This organization may be multiscaled, such as aggregates and agglomerates of platelets for alumina supports (Speyer *et al.*, 2020 ▸; Wang *et al.*, 2015 ▸), aggregates of cobalt nanoparticles for Fischer–Tropsch catalysts (Humbert *et al.*, 2018 ▸), or aggregates of sulfur slabs for hydrodesulfurization (HDS) catalysts (Humbert *et al.*, 2021 ▸). The particle-size distributions of the support or active phases are often well represented by a normal or lognormal distribution law depending on whether they are formed by coalescence or ripening (Granqvist, 1976 ▸; Datye *et al.*, 2006 ▸). Detailed characterization of the microstructure of heterogeneous catalysts is important to optimize their performance (activity, selectivity) (Munnik *et al.*, 2014 ▸).

Small-angle X-ray scattering (SAXS) probes the fluctuation of electron density in a material (Li *et al.*, 2016 ▸) at the nanometre scale. Therefore, it is well adapted to the characterization of heterogeneous catalysts. Using the anomalous mode (ASAXS) allows one to specifically probe the active phase (Haubold & Wang, 1995 ▸; Benedetti, 1997 ▸; Polizzi *et al.*, 2002 ▸). SAXS has already been widely employed for the characterization of colloidal suspension and materials like cement, metallic nanoparticles, oil, polymers, pharmaceuticals, food and proteins (Li *et al.*, 2016 ▸). SAXS relates the intensity *I* scattered at a wavevector 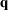 with the Debye correlation function or normalized covariance of the sample 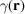 . Conventional data processing is carried out by splitting the scattered intensity into form and structure factors. Such an approach is very suitable for dilute suspensions, with the form factor being restricted to simple morphology and the structure factor being restricted generally to hard-core repulsion. However, for microstructures such as heterogeneous catalysts, conventional data processing may not be relevant. For instance, the multiscale models proposed by Beaucage (1995) ▸ give parameters that are easily interpretable only for fractal morphology, which is seldom encountered in heterogeneous catalysis. The program *MIXTURE* (Konarev *et al.*, 2003 ▸) fits the experimental scattering curve by modeling the multicomponent system with different form factors considering interparticle interactions. These interactions are described by structure factors calculated within the Percus–Yevick approximation for hard-sphere or sticky hard-sphere potential. However, the modeling of multiscale aggregation of particles is not obvious and gamma or lognormal particle-size distributions are not available. Another approach is proposed by Bressler *et al.* (2015*b*) ▸ with the *McSAS* software and gives a distribution of an arbitrary shape from a single form factor.

Exploitation of the SAXS intensity can also be performed in a less classical way thanks to Boolean models, either by direct calculation of 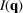 from a known covariance or by inverting the relation to extract 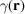 . The direct approach has been applied on a one-scale Boolean model of spheres (Gille, 2011 ▸), a union and intersection of Boolean models, a dead leaves model, a clipped Gaussian field model, and a Gaussian field intersection model (Gommes, 2018 ▸). The reverse approach has been applied for a one-scale Boolean model of spheres with monodisperse or exponential size distribution, Poisson polyhedra, the intersection of two Poisson polyhedron models, and the intersection of a Poisson polyhedron and the complement of a Poisson polyhedron (Sonntag *et al.*, 1981 ▸). Both approaches only require a single 1D Fourier transform and a numerical differentiation to obtain the SAXS intensity, which makes them computationally efficient. They are, however, restricted to models with known analytical covariance, namely spheres, and a very simple particle-size distribution (Sonntag *et al.*, 1981 ▸; Gille, 2011 ▸) or an arbitrary but discrete distribution (Gommes, 2018 ▸). Sorbier *et al.* (2019) ▸ proposed to extend this approach to multiscale Boolean models of spheres with arbitrary distribution laws, giving explicit formulae for lognormal, gamma and exponential distributions. Their work is, however, restricted to spherical particles and aggregates.

A matching correlation function does not unambiguously define a microstructure (Gommes *et al.*, 2012 ▸; Gommes, 2018 ▸). Yet, some parameters of Boolean models can be constrained either by knowledge of the synthesis process or from complementary characterization techniques, which can dramatically reduce the microstructures matching with a specified correlation function and is the strength of this kind of approach compared with conventional data processing.

The aim of this article is thus to extend the work of Gommes (2018) ▸ and Sorbier *et al.* (2019) ▸ to spheroidal grains and/or aggregates for interpretation of SAXS intensities by multiscale models. By multiscale we mean both the possibility to take into account aggregation of objects on very different size scales and allowing a size distribution of elementary objects or aggregates. Such an approach would enable the simulation of anisotropic morphology such as aggregation of platelets in an alumina support or aggregation of sulfur slabs in an HDS catalyst. In the limiting cases, it could approximate Boolean models of rods or thin discs for very high and very low aspect ratio of the spheroids, respectively. Section 2 recalls the basic equations for Boolean models and how to compute the SAXS intensity from the geometrical covariogram of the models. Section 3 is devoted to the calculation of the geometrical covariogram for a population of spheroids. In Section 4, we show results for one-scale Boolean models of spheroids and validate the approach with asymptotic models, isolated spheroids, isolated needles and isolated discs. Finally, Section 5 is devoted to an example of an application of a multiscale Boolean model of spheroids to the interpretation of SAXS data obtained on alumina catalyst supports.

## 2. Computation of scattered intensities and Boolean models

### 2.1. Computation of scattered intensities

For a known normalized covariance γ(*h*) the SAXS intensity reads (Levitz & Tchoubar, 1992 ▸)

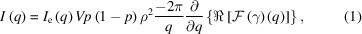

where *q* is the wavevector, *I*_e_(*q*) is the intensity scattered by one electron, *V* is the volume irradiated by the incident X-ray beam, 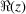 is the real part of *z*, *p* is the grain volume fraction and 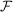 is the Fourier transform defined by 

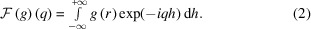

Equation (1) can be evaluated numerically by an efficient fast Fourier transform (FFT) algorithm. The scattering intensity for *q* → 0 is given by 

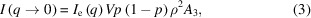

where *A*_3_ is the integral range of γ defined by 

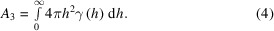




### 2.2. One-scale Boolean models

Boolean models are generated by sampling random Poisson points with a θ density and implanting on each Poisson point a random primary grain *A*′ (Matheron, 1967 ▸, 1975 ▸; Jeulin, 2022 ▸). The random Boolean set *A* is the union of the grains *A*′. Overlapping of the grains is allowed. We will refer hereafter to the case of a biphasic material where the solid fraction has a finite (ρ > 0) electronic density and the void fraction has a ρ = 0 electronic density, otherwise known as a porous material. The approach can, however, be easily extrapolated to materials with more than two phases.

The geometrical covariogram of the grain *A*′ completely defines one-point (porosity or solid fraction) and two-point statistics (void–void, void–solid and solid–solid covariances). The geometrical covariogram *K*(**h**) is defined as the expectation E of the volume of the intersection between the grain *A*′ and *A*′_**h**_, the grain translated by a vector **h**. In the following we will restrict ourselves to isotropic models where *K* only depends on *h* = *∥* **h** *∥*: 

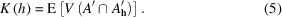

The grain volume fraction *p* of a Boolean set is related to the intensity of the process θ and to *K* by 

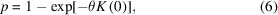

where *K*(0) is the expectation of the volume of the primary grains. The porosity of the model ɛ = 1 − *p* is trivially related to the volume fraction *p*. The two-point correlation function of the set (of the solid phase) *C*_11_(*h*), the two-point correlation function of the complement of the set (of the void phase) *C*_00_(*h*) and the normalized covariance γ(*h*) are related by 

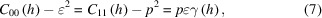

where the normalized covariance reads (Gommes, 2018 ▸; Sorbier *et al.*, 2019 ▸)

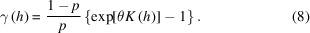

From the normalized covariance, it is possible to compute the specific surface area *S*_*V*_ by (Debye *et al.*, 1957 ▸)

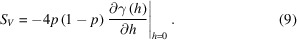




### 2.3. Union and intersection of models

Union and intersection of Boolean models allow one to produce a grain-size distribution (union) or multiscale models (intersection) while keeping closed analytical formulae.

The porosity or void–void two-point correlation function of the union of Boolean models is the product of the porosity or void–void correlation functions of each model. The solid fraction or solid–solid two-point correlation function of the intersection of Boolean models is the product of the solid fraction or solid–solid correlation functions of each model (Gommes, 2018 ▸; Sorbier *et al.*, 2019 ▸). A one-scale Boolean model with a size and shape distribution of grains may be viewed as a union of Boolean models of these grains having a specific size and shape. If *K*(*h*, *R*, λ) is the geometrical covariogram of a grain of size *R* and aspect ratio λ, and *P*(*R*, λ) is the size and aspect-ratio distribution of the grains, the geometrical covariogram of the set reads

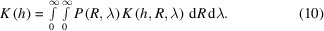

The calculation of *K*(*h*, *R*, λ) allows one to compute the scattered intensities for the intersection and union of Boolean models of grains with a size and shape distribution from equations (1), (8) and (10).

## 3. Geometrical covariogram of spheroids

In this section, we consider spheroids as ellipsoids with semi-axes (*R*, *R*, λ*R*). Such a solid has a gyration radius of 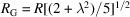 .

### 3.1. Single size

**Figure 1:**
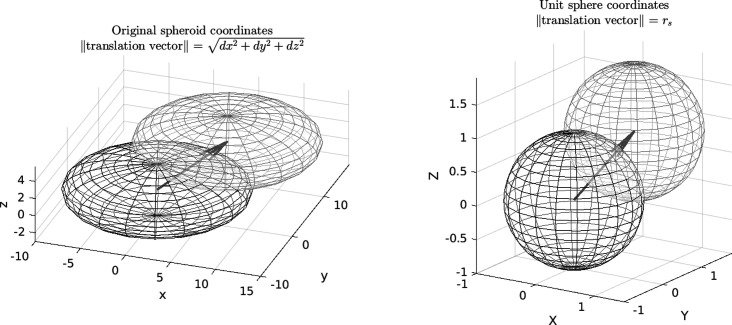
Coordinate change to transform a spheroid into a unit sphere, where *r*_s_ is the norm of the translation vector in the new coordinates.

In this subsection, the geometrical covariogram of single-size spheroids is calculated, *i.e* *K*(*h*, *R*, λ) in equation (10). To this end, a change of coordinates is applied so as to transform the spheroid into a unit sphere, the covariogram of which is known (see Fig. 1 ▸):

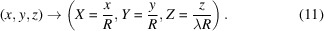

In these new coordinates the translation vector **H** is 

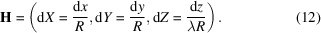

The norm of the translation vector is denoted as *r*_s_ = *∥* **H** *∥*. It is given by 

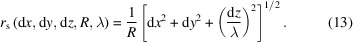

Now, the volume *V*_s_ of the intersection of two unit spheres distant by *r*_s_ = *∥*(d*X*, d*Y*, d*Z*)*∥* is (Guinier & Fournet, 1955 ▸) 

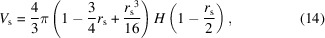

where *H* is the Heaviside step function.

Hence the volume *V*(d*x*, d*y*, d*z*) of the intersection between the spheroid and its translation by the vector (d*x*, d*y*, d*z*) reads

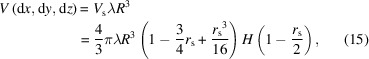

where *r*_s_ is defined by equation (13).

**Figure 2:**
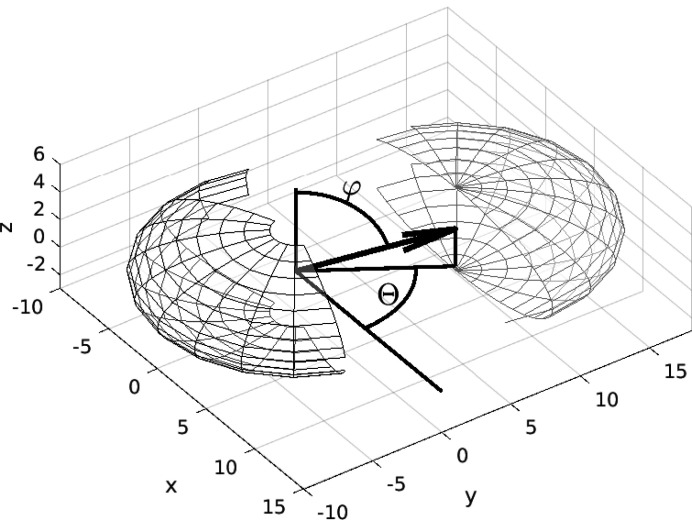
Spherical coordinates of the translation vector (original coordinates).

It remains to calculate the expectation of the volume *V*(d*x*, d*y*, d*z*) for all possible translation directions [see equation (5)]. If the translation vector is expressed in spherical coordinates (see Fig. 2 ▸) as

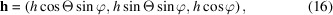

we have 

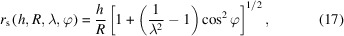

where *r*_s_ does not depend on Θ. This is due to the symmetry of the spheroid with respect to Θ (the spheroid is a sphere elongated or compressed along the *z* direction). The geometrical covariogram reads

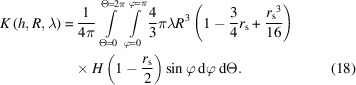

The integration over Θ is trivial (*r*_s_ does not depend on θ) and the integration over φ may be reduced to the 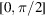 range due to the symmetry *r*_s_(π − φ) = *r*_s_(φ) and 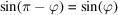 , such that 

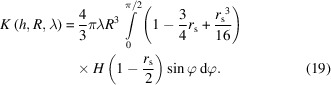




### 3.2. Size distribution

In the following we will suppose that the spheroidal grains have a size distribution of *P*(*R*) but a constant aspect ratio of λ. From equation (10), the geometrical covariogram reads

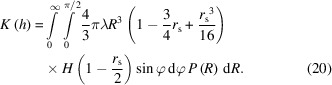

Let us define 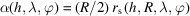 . The symbol α does not depend on *R*. Let us also remark that 

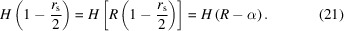

After changing the order of integration with respect to φ and *R*, we have 

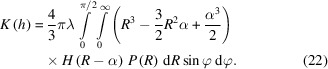

Since *H*(*R* − α) is equal to 0 for *R* < α and equal to 1 for *R* ≥ α, and since α does not depend on *R*, this expression can be rewritten as 

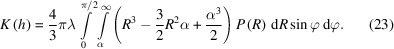

 If we define 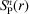 , the partial uncentered moment of order *n* of *P*, by

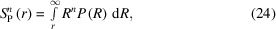

then equation (23) can be expressed with these uncentered moments as

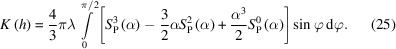

Provided that the *P* distribution allows one to evaluate its partial moments in a closed analytical form, equation (25) can be evaluated numerically. Partial moments can be analytically evaluated for monomodal, exponential, gamma, lognormal (Sorbier *et al.*, 2019 ▸) and normal distribution laws, for example.

### 3.3. Implementation in *plug im!* module

Equations (25), (8) and (1) have been implemented in Fortran code and provided through a module called *SAXSBME* for the *plug im!* platform (Moreaud, 2018 ▸). The integral in equation (25) is numerically evaluated by the *QUADPACK* routines of the *SLATEC* library (Piessens *et al.*, 1983 ▸). Intersections and unions of Boolean models of ellipsoids on up to three scales are provided. Monomodal, exponential, gamma and lognormal size distribution laws are implemented with independent parameters for each scale. The aspect ratio of ellipsoids is kept constant for each scale. Equation (1) is implemented using the FFT routines of the Intel Math Kernel Library. The sampling of the covariance γ(*h*) is performed in direct space with 2^*n*^ points equally spaced from 0 up to 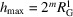 , where 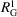 is the expectation of the gyration radius of the larger scale. Parameters *n* and *m* control the minimum and maximum scattered wavevectors (*q*_min_ and *q*_max_), the sampling step in *q* space (Δ*q*), and the computation times. The minimum and maximum scattered wavevectors are calculated as

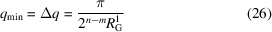

and

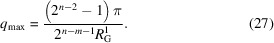

Use of parameters *n* = 22 and *m* = 12 leads to computation times in the order of a second on a standard laptop computer (Intel Core i5-8250U, 8 GB RAM).

## 4. Validation on diluted models and one-scale Boolean models

The asymptotic case of θ → 0 yields isolated objects for which analytical formulas are known or are numerically implemented in independent software.

### 4.1. Isolated spheroids

**Figure 3:**
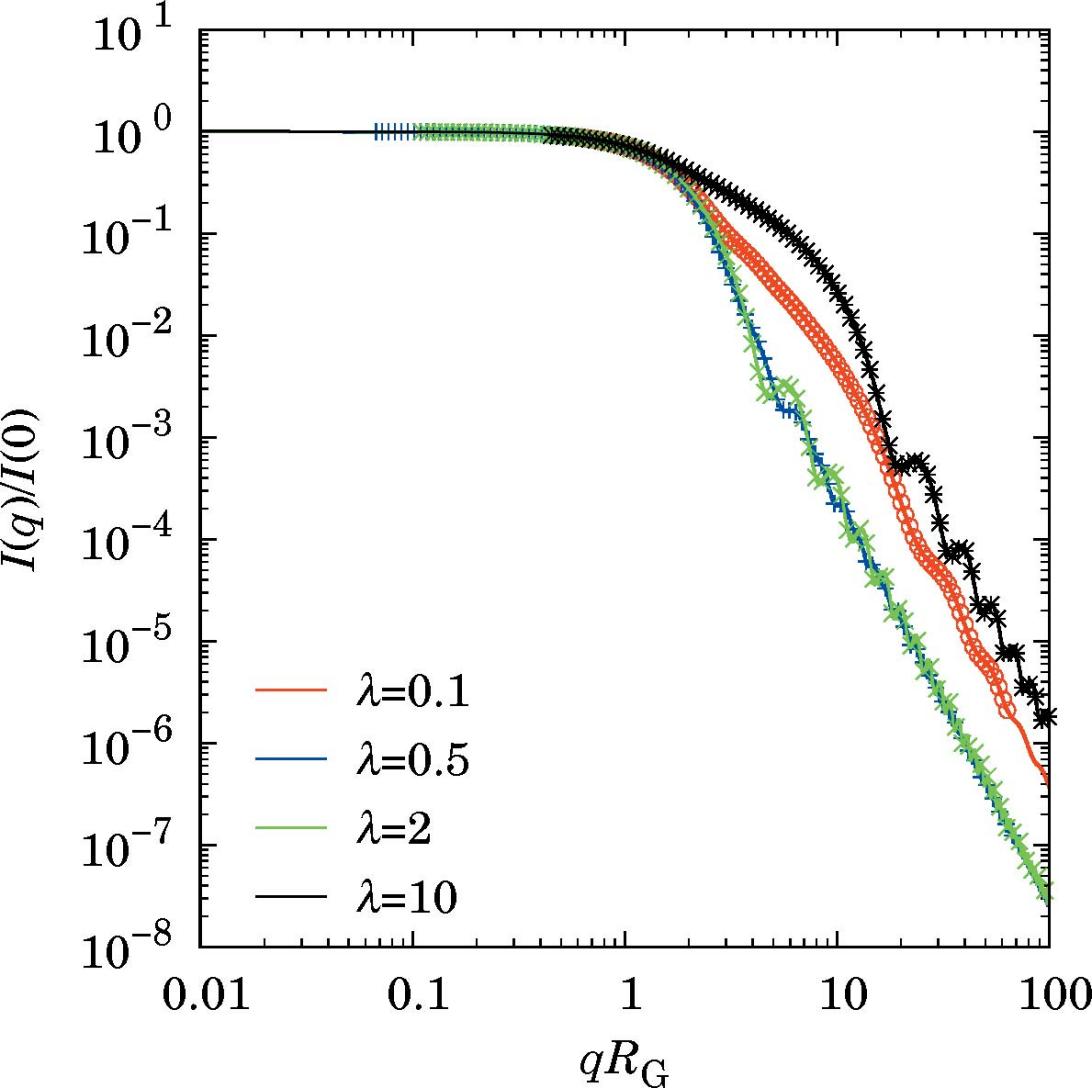
Scattered intensities from isolated spheroids. The lines are from *SASFit* computation and the symbols are numerical computation from this work.

The *SASFit* software (Breßler *et al.*, 2015*a* ▸; Kohlbrecher & Breßler, 2022 ▸) allows the calculation of scattered intensity for isolated spheroids. Fig. 3 ▸ compares the values of scattered intensities from the reference *SASFit* software with those of this work. A perfect agreement is found for any aspect ratio.

### 4.2. Isolated rods and discs

The formula for scattered intensity *I*_D_(*q*) from infinitely thin discs of radius *R* [gyration radius *R*/(2)^1/2^] is already known (Guinier & Fournet, 1955 ▸):

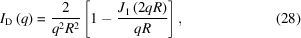

where *J*_1_ is the first-order Bessel function of the first kind. Thin discs are the limiting case of a spheroid for λ → 0. The scattered intensity of infinitely thin rods of length 2λ*R* (gyration radius λ*R*/3) is also known:

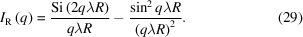

**Figure 4:**
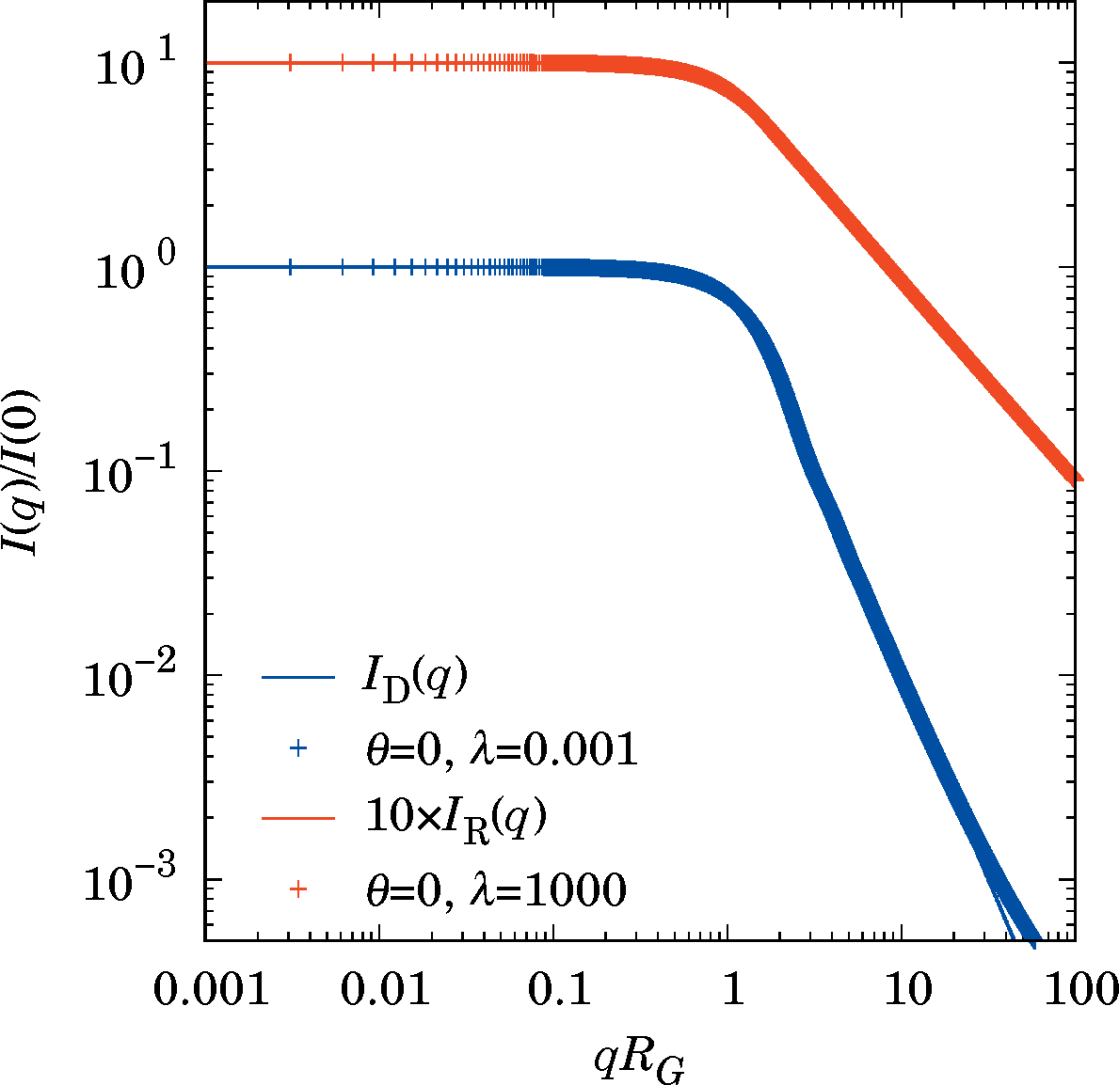
Scattered intensities from isolated discs (blue) and rods (red). The lines are analytical formulae (28) and (29), and the symbols are numerical computation from this work.

Thin rods are the limiting case of a spheroid for λ → ∞. Fig. 4 ▸ shows a comparison of equations (28) and (29) with a numerical computation (λ = 0.001 for discs and λ = 1000 for rods). For low *q* (large scale) the agreement is very good, whereas discrepancies are found at high *q* (small scale) due to non-asymptotic values of λ. These results indicate that there are no numerical problems even for very high or very low aspect ratio.

### 4.3. One-scale Boolean models of spheroids

**Figure 5:**
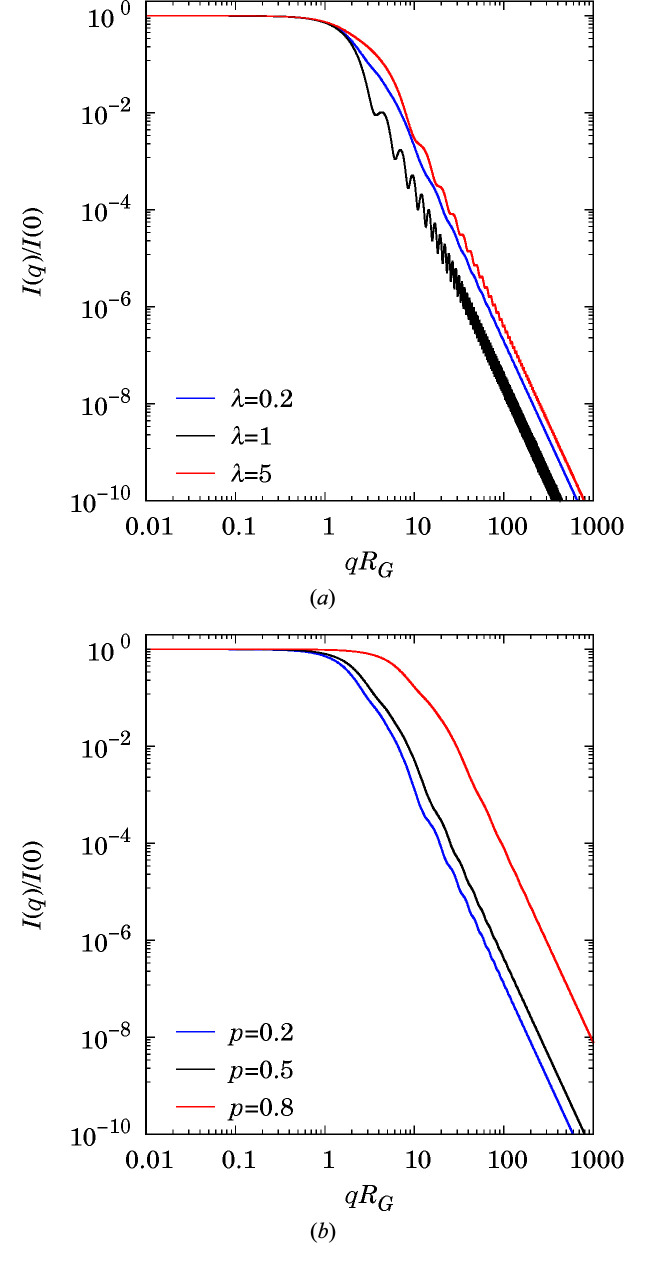
A one-scale Boolean model of spheroids with monomodal size distribution *R* = 10. (*a*) For *p* = 0.5 and varying aspect ratio λ. (*b*) For λ = 0.2 and varying volume fraction *p*. *R*_G_ is the gyration radius of the primary grain.

**Figure 6:**
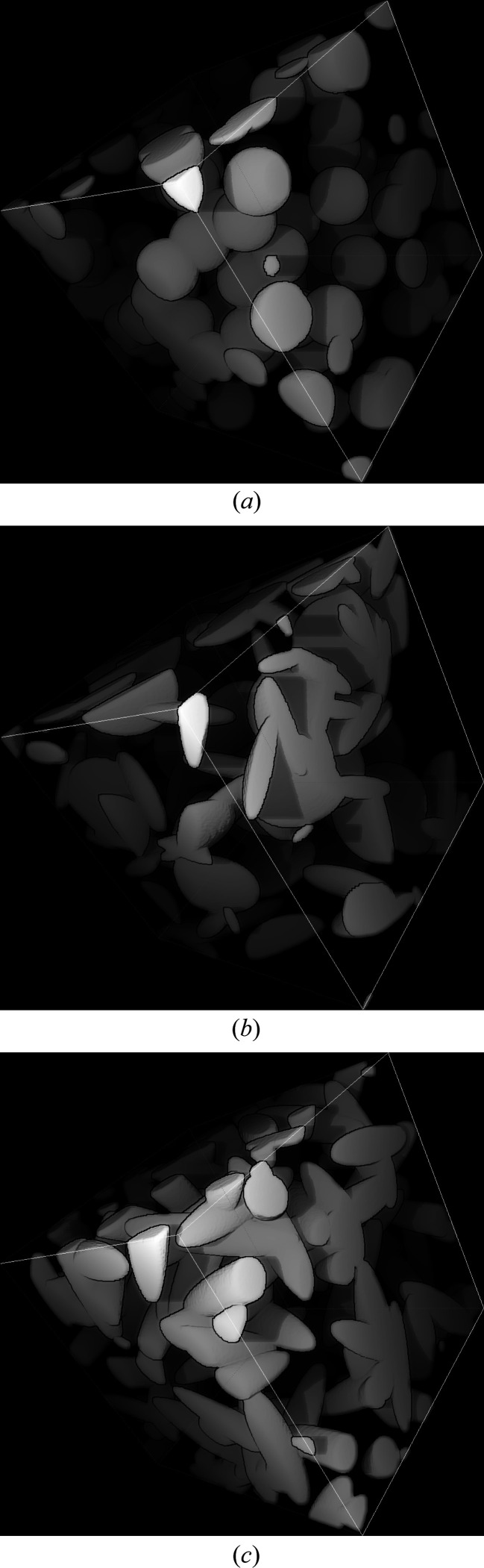
A one-scale Boolean model of spheroids with monomodal size distribution, *p* = 0.2 and varying aspect ratio: (*a*) λ = 1, (*b*) λ = 0.2 and (*c*) λ = 5.

Fig. 5 ▸ shows some simulated intensities for a one-scale Boolean model of spheroids with monomodal size distribution. The case λ = 1 corresponds to a one-scale Boolean model of spheres. At a fixed volume fraction *p*, oscillations are more marked for spheres and prolate spheroids (λ > 1) than for oblate spheroids (λ < 1). At a fixed aspect ratio λ, the higher the volume fraction, the less pronounced the oscillations. One realization for each model is illustrated in Fig. 6 ▸.

## 5. Application to the characterization of alumina catalyst supports

To highlight the interest of multiscale Boolean models of spheroids, in this section we show how they can be used to interpret SAXS data of alumina catalyst supports. Two different samples are considered in this work: boehmite, and γ-alumina obtained by calcining this boehmite. Their textural properties obtained from nitrogen physisorption and helium pycnometry are detailed in Table 1 ▸. The first sample is an extrudate of boehmite AlOOH. The raw boehmite was an industrial product, provided by Axens (https://www.axens.net), prepared by precipitation from aluminium sulfate and sodium aluminate solutions. It was shaped by kneading extrusion, and finally dried at 80°C. The alumina catalyst was obtained by calcining the boehmite extrudates in an oven at 540°C. Catalyst supports are used as pellets in industrial reactors. In addition to verifying the potential of this modeling approach to simulate SAXS curves, we will take the opportunity to visualize the effect of calcination on the multiscale arrangement of boehmite particles. Boehmite particle aggregates present an elongated shape as observed by scanning transmission electron microscopy (STEM) (Ferri *et al.*, 2022 ▸). Therefore, an ellipsoidal morphology makes sense to describe these aggregates of particles.

### 5.1. SAXS data acquisition

**Figure 7:**
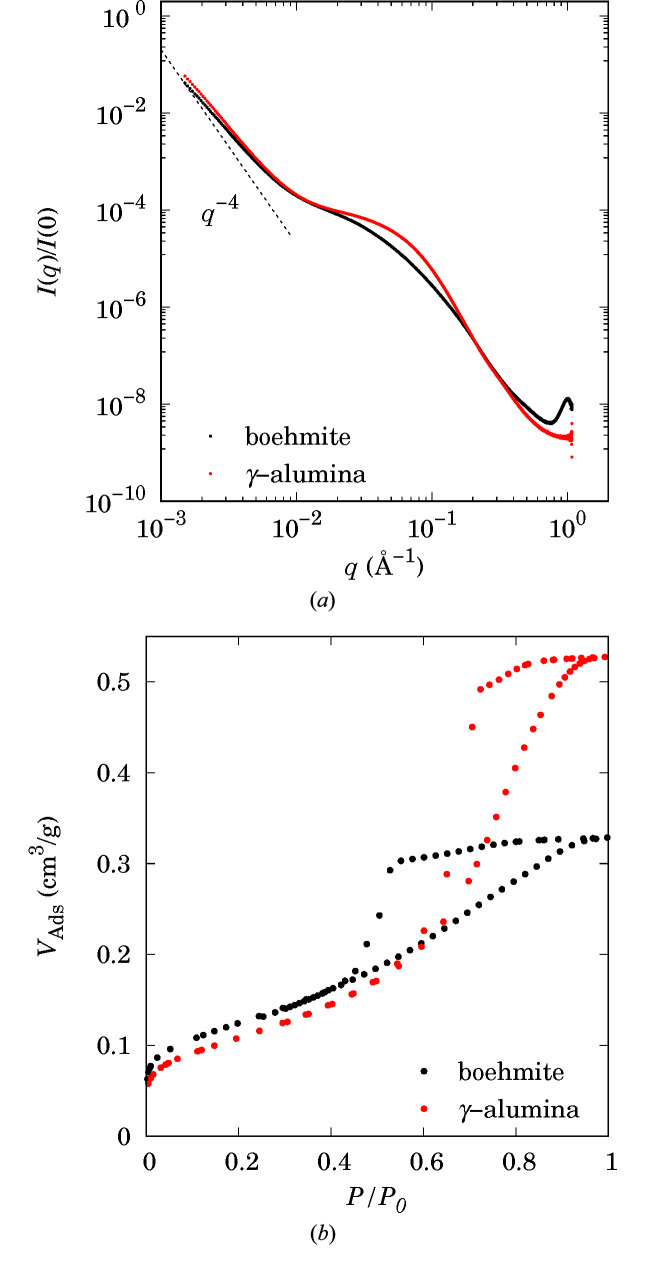
(*a*) Experimental SAXS data for boehmite and γ-alumina. Intensities are normalized by *I*(0) and the dashed line is a guide representing the Porod regime (*q* ^−4^). (*b*) Nitrogen physisorption data for boehmite and γ-alumina, where *V*_Ads_ is the volume adsorbed and *P* refers to pressure.

SAXS was carried out on the SWING beamline of synchrotron SOLEIL (Saclay, France) with a 12 keV incident beam. Boehmite and alumina extrudates were first crushed to obtain fine powders, before being pelleted (thickness 0.2 mm) for analysis. In order to cover a wider range of *q* values, three sample–detector distances (1, 3 and 6.7 m) were employed. The scattering images were recorded using an AVIEX PCCD170170 detector. To increase the statistics of the scattering intensity, ten images were acquired at each detector–sample configuration. Through 1D reduction, raw data were corrected with respect to acquisition time, geometrical effects like the projection of the detector plane on the sphere with radius equal to the sample–detector distance and the incoming photon flux, and then averaged to increase the statistics. Finally, in order to calibrate the intensity in absolute units, *i.e.* expressed as differential scattering cross section per unit volume in cm^−1^, glassy carbon was measured, and a correction factor was calculated from the ratio between the theoretical data and the experimental data. Experimental SAXS data are plotted for boehmite and alumina in Fig. 7 ▸(*a*) and nitrogen sorption data are plotted in Fig. 7 ▸(*b*).

### 5.2. Fit on multiscale Boolean model of spheroids

**Figure 8:**
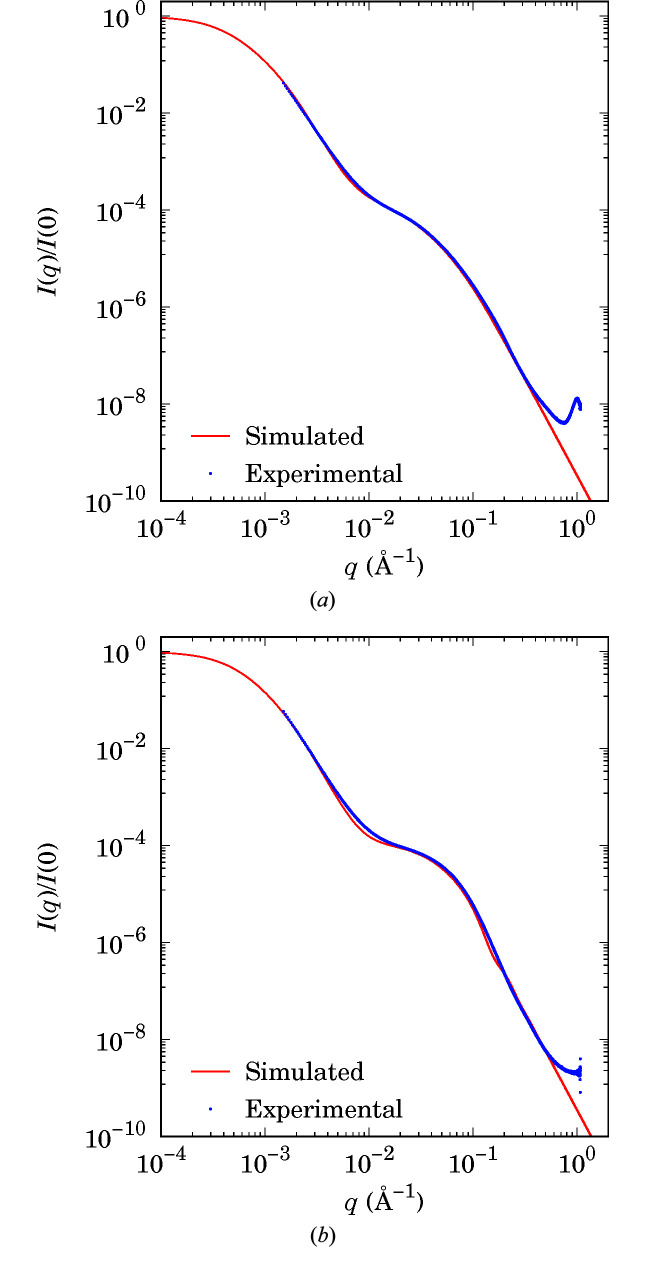
Experimental SAXS data (symbols) for (*a*) boehmite and (*b*) γ-alumina, and simulated SAXS data (curves) obtained from multiscale Boolean models of spheroids. Intensities are normalized by *I*(0).

**Figure 9:**
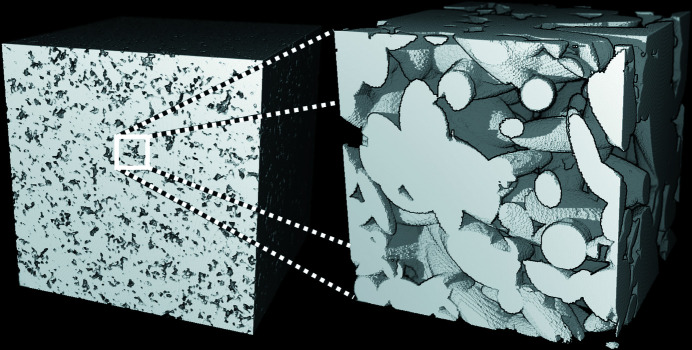
Simulation of boehmite with a Boolean model according to the parameters in Table 2 ▸: left agglomerate scale (size 300 nm^3^, 0.3 nm voxel^−1^) and right aggregate scale (size 30 nm^3^, 0.1 nm voxel^−1^). Agglomerates are too large to be seen at these scales.

The experimental data can successfully be fitted with SAXS simulated curves obtained from two significantly different multiscale Boolean models [see Figs. 8 ▸(*a*) and 8 ▸(*b*)]. These models are represented in Figs. 9 ▸ and 10 ▸, which are further discussed in Section 5.5 to highlight the effect of calcination. Three scale levels are necessary to describe the boehmite and alumina structures, as already seen in the literature from STEM images (Speyer *et al.*, 2020 ▸): the scales of particles, aggregates and agglomerates. Hence, the models *M* have been built by means of the intersection of three Boolean models, *i.e.* *M* = *A* ∩ *B* ∩ *C*, where *A*, *B* and *C* are Boolean models of spheroids accounting for the scales of particles, aggregates and agglomerates, respectively. Each scale has its own aspect ratio λ, volume fraction *p*, and parameters μ and σ for modeling its size distribution by a lognormal law. All parameters were fitted, with the only constraint being that the mean size of agglomerates is larger than that of aggregates whose mean size is larger than that of particles. The complexity of the models was fixed not from the shape of the SAXS intensities but from the boehmite and alumina structures as described in the literature (Speyer *et al.*, 2020 ▸). The parameters of the model are reported for both samples in Table 2 ▸. From the scale parameter μ and the shape parameter σ of the lognormal law, it is possible to calculate the mean radius (in volume) of the ellipsoids *R*_V_ and the corresponding gyration radius *R*_G_ for the three different scales by the following formulae: 

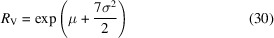

and 

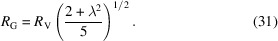

Furthermore, the specific surface areas and the porous volumes have been calculated from equations (9) and (6) and experimental values of structural densities. Due to the lack of data at *q* < 10^−3^ Å^−1^, the parameters of the agglomerates scale are very uncertain. For the boehmite model, the platelets are modeled as flat ellipsoids that are twice as large as they are thick and aggregates are modeled as elongated grains that are 3.5 times as long as they are large. For both samples, the scale that contributes most to the scattering (*p* closest to 0.5) is the aggregate scale made of elongated grains.

### 5.3. Comparison with nitrogen physisorption

No adequate analytical SAXS model allows one to correctly describe the microstructure of alumina as the structure is very complex due to the calcination effect. However, the specific surface and the porous volume can be compared with those measured by nitrogen physisorption on extrudates. Importantly, the two porous volumes must be compared with caution. The nitrogen physisorption volume leads to a mesoporous volume, considering pore sizes between 2 and 50 nm. In comparison, the total porous volume calculated from the Boolean model is the sum of micro-, meso- and macro-porous volume, from ∼2 to ∼300 nm. The two are not directly comparable. Thus, it seems more accurate to compare the nitrogen physisorption porosity with the simulated intra- and inter-aggregate porosity *p*_1_(1 − *p*_2_ *p*_3_), excluding the inter-agglomerate porosity (1 − *p*_1_). The simulated specific surface areas, 304 and 230 m^2^ g^−1^ for boehmite and alumina, respectively, are comparable to those obtained by nitrogen physisorption, 288 and 252 m^2^ g^−1^, respectively. Considering the uncertainties on the structural density (2.8 and 3.2 g cm^−3^) and on the nitrogen physisorption values, the simulated values are in good agreement with experimental ones. Concerning the mesoporous volume, 0.35 and 0.53 ml g^−1^, respectively, they are also in good agreement with those measured by nitrogen physisorption (0.33 and 0.53 ml g^−1^, respectively).

### 5.4. Comparison with other SAXS data interpretation

Traditionally, absolute-scale measurements allow one to calculate specific surface area from the Porod slope at large *q* and the porosity from the whole curve. However, for multiscale material such as boehmite and alumina, the SAXS signal takes into account all the kinds of porosity (microporosity, mesoporosity and part of macroporosity) in the *q* range of measurement. Hence, it is difficult to decorrelate all the contributions and the calculated porosity cannot be directly compared with the nitrogen physisorption. Moreover, in boehmite and alumina, the quantity of adsorbed water mol­ecules can significantly vary, which can impact appreciably the electronic density of the material and so the calculation of porosity and specific surface area. Besides, traditional data interpretation is unable to provide more information such as object anisotropy or the width of their size distribution. Data processing using the Beaucage model (Beaucage, 1995 ▸) is only possible for boehmite data (Speyer *et al.*, 2020 ▸). This approach has been applied to the boehmite sample and has led to the estimation of various parameters: the gyration radius of the particles 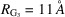 , the aggregates’ gyration radius 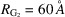 , the aggregates’ mass fractal dimension 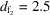 , the agglomerates’ gyration radius 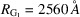 and the agglomerates’ fractal dimension 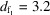 , which corresponds to a surface fractal dimension of 2.8 for dense objects (Speyer *et al.*, 2020 ▸). Compared with the Beaucage model value, the simulated particle gyration radius is of the same order of magnitude but quite a bit higher. However, the crystallite gyration radius estimated by the Beaucage model is quite uncertain as the curve inflection is not significant in the *q* range corresponding to the crystallites, around 2 × 10^−1^ Å^−1^. Nonetheless, the gyration radius estimated from the Beaucage model and the simulated ones, for both aggregate and agglomerate scales, are in very good agreement. The simulated 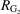 and 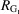 , 56 and 2324 Å, respectively, are comparable to the estimated values of 60 and 2560 Å, respectively, from the Beaucage model. Finally, the simulated aspect ratios of the aggregates and agglomerates can be compared with the fractal dimensions obtained by the Beaucage model. The fractal dimension of the aggregates is found to be 2.5, which is representative of a moderately elongated structure, as a dimension of 1 corresponds to a fiber morphology and 3 to a sphere (Schaefer & Hurd, 1990 ▸). However, the dimension of the agglomerates is higher than 3, which indicates dense and almost spherical objects. The simulated ellipsoidal morphologies correctly convey these characteristics: ellipsoids related to aggregates are more elongated, with λ_2_ = 3.5, whereas agglomerates are better represented with spheres (λ_1_ = 1). To conclude, spheroidal Boolean models are well adapted to boehmite and alumina microstructure, and allow one to simulate SAXS and nitrogen physisorption data comparable to the experimental data.

### 5.5. Effect of calcination on the microstructure

As observed in Figs. 9 ▸ and 10 ▸, the microstructure before and after calcination is significantly different. The agglomerate scale is not modified by the calcination. The aggregate scale is deeply altered, resulting in a change of porous volume and specific surface area. In fact, during calcination, the aggregates are densified, as their porosity decreases from 7 to 0% due to the crystallite sintering phenomenon, which leads to a decrease of the specific surface area. This densification is accompanied by crystallographic transformation and dehydration (from AlOOH to γ-Al_2_O_3_), also resulting in a decrease of material volume fraction at the agglomerate scale *p*_2_ *p*_3_, from 0.50 to 0.37. The ratio between these two values, 1.36, is directly comparable with the theoretical ratio between the AlOOH and γ-Al_2_O_3_ molar volumes (1.34). The size of aggregates also reduces, with the gyration radius decreasing from 56.3 to 40.2 Å, even more than the size decrease expected from the volume fraction ratio. It can be assumed that fragmentation phenomena also occur during calcination, which could explain the significant decrease in aggregate size. All these modifications are well illustrated in Figs. 9 ▸ and 10 ▸, and explain the increase of the porous volume.

## 6. Conclusions

Numerical computation of scattered intensities of multiscale Boolean models of spheroids allows one to take into account anisotropic primary grains such as alumina platelets or sulfur slabs. We provide a Fortran implementation via the *plug im!* platform. The code has been validated by considering analytical formulae and results from the *SASFit* software in the asymptotic case of diluted models (isolated grains). Implementation is efficient enough to compute scattered intensities on timescales of the order of a second. We have shown how such an approach may help to interpret SAXS data acquired on alumina catalyst supports. It has allowed us to interpret the experimental data through geometrical parameters such as the volume fraction, radius and elongation of particles, aggregates, and agglomerates. The comparison of results obtained on boehmite and on γ-alumina, before and after calcination, highlighted the major modifications due to sintering, crystallographic, dehydration and fragmentation phenomena. Such a study would not have been possible without the use of morphological models and the computation of their SAXS diagram. Moreover, morphological models make it possible to compute other morphological properties such as specific surface area, nitrogen physisorption isotherm (Hammoumi *et al.*, 2022 ▸), geometrical tortuosity (Chaniot *et al.*, 2019 ▸) and diffusion tortuosity factor (Wang *et al.*, 2017 ▸). 

## Figures and Tables

**Figure 10:**
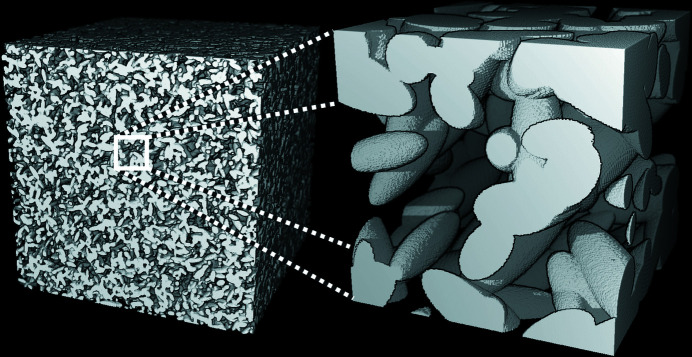
Simulation of γ-alumina with a Boolean model according to the parameters in Table 2 ▸: left agglomerate scale (size 300 nm^3^, 0.3 nm voxel^−1^) and right aggregate scale (size 30 nm^3^, 0.1 nm voxel^−1^). Agglomerates are too large to be seen at these scales.

**Table 1:** Textural properties of catalyst supports

Sample	Specific surface area *S*_BET_ (m^2^ g^−1^)	Porous volume *V*_P_ (cm^3^ g^−1^)	Structural density ρ_S_ (g cm^−3^)
Boehmite AlOOH	288	0.33	2.8
γ-Alumina Al_2_O_3_	252	0.53	3.2

**Table 2:** Parameters of the intersection *M* = *A* ∩ *B* ∩ *C* of Boolean models of spheroids representative of the boehmite and γ-alumina samples and induced textural properties

Scale	Fit parameters	Boehmite	γ-Alumina
Particles *A*	μ_3_ (Å)	3.46	–
	σ_3_ (Å)	0.1	–
	λ_3_	0.55	–
	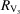 , 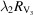 (Å)	33, 18	–
	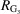 (Å)	22	–
	*p*_3_	0.93	1
Aggregates *B*	μ_2_ (Å)	2.7	3.22
	σ_2_ (Å)	0.48	0.15
	λ_2_	3.5	3
	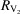 , 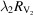 (Å)	33, 117	27, 81
	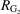 (Å)	56.3	40.2
	*p*_2_	0.54	0.37
Agglomerates *C*	μ_1_ (Å)	7.2	7.2
	σ_1_ (Å)	0.48	0.45
	λ_1_	1	1
	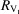 , 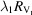 (Å)	3000, 3000	2720, 2720
	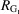 (Å)	2324	2110
	*p*_1_	0.94	0.92
Total *M*	*S*_BET_ (m^2^ g^−1^)	304	230
	ε_meso_ = *p*_1_(1 − *p*_2_ *p*_3_)	0.47	0.58
	ε = 1 − *p*_1_ *p*_2_ *p*_3_	0.53	0.66
	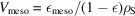 (cm^3^ g^−1^)	0.35	0.53
	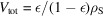 (cm^3^ g^−1^)	0.40	0.61
